# Subtype-specific accumulation of intracellular zinc pools is associated with the malignant phenotype in breast cancer

**DOI:** 10.1186/s12943-015-0486-y

**Published:** 2016-01-05

**Authors:** Paige Chandler, Bose S. Kochupurakkal, Samina Alam, Andrea L. Richardson, David I. Soybel, Shannon L. Kelleher

**Affiliations:** The Interdisciplinary Graduate Program in Physiology, Penn State Hershey College of Medicine, Hershey, PA 17033 USA; The Department of Cellular and Molecular Physiology, Penn State Hershey College of Medicine, Hershey, PA 17033 USA; The Department of Pharmacology, Penn State Hershey College of Medicine, Hershey, PA 17033 USA; The Department of Surgery, Penn State Hershey College of Medicine, Hershey, PA 17033 USA; Dana Farber Cancer Institute, Boston, MA 02115 USA; Department of Pathology, Brigham and Women’s Hospital, Harvard Medical School, Boston, MA 02115 USA

**Keywords:** Breast cancer, Basal/Luminal, Metallothionein, Zinc, Zinc importer, Zinc transporter

## Abstract

**Background:**

Zinc (Zn) hyper-accumulates in breast tumors and malignant cell lines compared to normal mammary epithelium. The mechanisms responsible for Zn accumulation and the consequence of Zn dysregulation are poorly understood.

**Methods:**

Microarrays were performed to assess differences in the expression of Zn transporters and metallothioneins (MTs) in human breast tumors and breast cancer cell lines. Real-time PCR and immunoblotting were employed to profile Zn transporter expression in representative luminal (T47D), basal (MDA-MB-231), and non-malignant (MCF10A) cell lines. Zn distribution in human tumors was assessed by X-ray fluorescence imaging. Zn distribution and content in cell lines was measured using FluoZin-3 imaging, and quantification and atomic absorption spectroscopy. Functional consequences of ZnT2 over-expression in MDA-MB-231 cells including invasion, proliferation, and cell cycle were measured using Boyden chambers, MTT assays, and flow cytometry, respectively.

**Results:**

Gene expression profiling of human breast tumors and breast cancer cell lines identified subtype-specific dysregulation in the Zn transporting network. X-ray fluorescence imaging of breast tumor tissues revealed Zn hyper-accumulation at the margins of Luminal breast tumors while Zn was more evenly distributed within Basal tumors. While both T47D and MDA-MB-231 cells hyper-accumulated Zn relative to MCF10A cells, T47D cells accumulated 2.5-fold more Zn compared to MDA-MB-231 cells. FluoZin-3 imaging indicated that Zn was sequestered into numerous large vesicles in T47D cells, but was retained in the cytoplasm and found in fewer and larger, amorphous sub-cellular compartments in MDA-MB-231 cells. The differences in Zn localization mirrored the relative abundance of the Zn transporter ZnT2; T47D cells over-expressed ZnT2, whereas MDA-MB-231 cells did not express ZnT2 protein due to proteasomal degradation. To determine the functional relevance of the lack of ZnT2 in MDA-MB-231cells, cells were transfected to express ZnT2. ZnT2 over-expression led to Zn vesicularization, shifts in cell cycle, enhanced apoptosis, and reduced proliferation and invasion.

**Conclusions:**

This comprehensive analysis of the Zn transporting network in malignant breast tumors and cell lines illustrates that distinct subtype-specific dysregulation of Zn management may underlie phenotypic characteristics of breast cancers such as grade, invasiveness, metastatic potential, and response to therapy.

**Electronic supplementary material:**

The online version of this article (doi:10.1186/s12943-015-0486-y) contains supplementary material, which is available to authorized users.

## Background

Breast cancer is a heterogeneous disease at the molecular, histopathological, and clinical level. Through gene expression profiling, four subtypes based on expression of estrogen receptor (ER), progesterone receptor (PR), and epidermal growth factor receptor 2 (HER2) are recognized including: Luminal A (ER^+^/PR^+^/HER2^−^), Luminal B (ER^+^/PR^+^/HER2^+^), Basal (ER^−^/PR^−^/HER2^−^) and HER2- enriched (ER^−^/PR^−^/HER2^+^). These subtypes differ in incidence [1], aggressiveness, and response to therapy [2, 3]. Recently, it has been reported that breast tumors accumulate zinc (Zn) to levels well above those observed in normal tissue [4]. The degree of Zn accumulation is associated with cancer progression [5] and malignancy [6]. However, the mechanisms responsible for Zn accumulation, and the relationship between Zn accumulation and breast cancer subtype are not understood.

A multitude of cellular processes are regulated by Zn including transcription, cell signaling, proliferation, invasion, apoptosis, and autophagy [7]. Cellular Zn metabolism is tightly regulated by a “Zn transporting network” which consists of 24 Zn transporting proteins that transport Zn into discrete sub-cellular compartments. The ZnT family of Zn transporters (*SLC30A1-10* gene family) contains 10 members (ZnT1-10) [8] that export Zn from the cytoplasm, either directly across the cell membrane or into intracellular compartments. The ZIP family of Zn transporters (*SLC39A1-14* gene family) contains 14 members (ZIP1-14) [9] and facilitates Zn import into the cytoplasm, either from across the cell membrane or from within a sub-cellular compartment. Cellular Zn management is also regulated by metallothioneins (MTs) [10], which are Zn binding proteins that buffer cytoplasmic Zn. ZnT2-mediated Zn accumulation into vesicles and MT-binding are the two primary mechanisms through which cells protect themselves from Zn toxicity, and both are positively regulated by Zn exposure through the activation of four metal responsive elements (MREs) in their promoters [11, 12].

Over-expression of several Zn transporters (ZIP6, ZIP7, ZIP10, and ZnT2) [13–19] is associated with Zn hyper-accumulation in breast tumors and several breast cancer cell lines. ZIP6 over-expression has been noted in ER^+^ subtypes [14] and is associated with less aggressive tumors [14]. Similarly, ZnT2 over-expression accumulates Zn in vesicles which protects ER^+^ T47D cells from Zn toxicity [18]. In contrast, ZIP10 is over-expressed in highly invasive, basal-like cell lines (MDA-MB-231 and MDA-MB-435S cells) and potentiates invasion [13]. Similarly, ZIP7 over-expression in tamoxifen-resistant MCF7 cells is associated with enhanced motility [20]. In addition to Zn transporters, MT over-expression is documented in ~88 % of invasive ductal carcinoma tissue biopsies [21], and is generally associated with poor prognosis [22] and high histological grade [21]. However, reports of Zn transporter dysregulation are sporadic and a comprehensive analysis of Zn management in specific breast cancer subtypes has not been reported.

We reasoned that the molecular portrait of the Zn transporting network may be very different between malignant subtypes, and perhaps even a driver of their phenotypic behaviors. Herein, we used targeted genomic, proteomic, and Zn profiling in breast tumors and malignant cell lines that have characteristic features of Luminal (low-invasive, ER^+^/PR^+^/HER2^−^; T47D cells) and Basal (highly invasive, ER^−^/PR^−^/HER2^−^; MDA-MB-231 cells) subtypes. We observed subtype-specific differences in Zn management between Luminal and Basal breast tumors, and in cell culture models of luminal and basal-like breast cancer cells. Importantly, we found that Zn sequestration in vesicles through expression of ZnT2 profoundly reduced the proliferative and invasive phenotype of MDA-MB-231 cells, indicating that Zn dysregulation is subtype-specific, which may inform the development of novel diagnostic or therapeutic strategies.

## Results

### The distribution of Zn accumulation in breast tumors differs between Luminal and Basal tumors

**Fig. 1:**
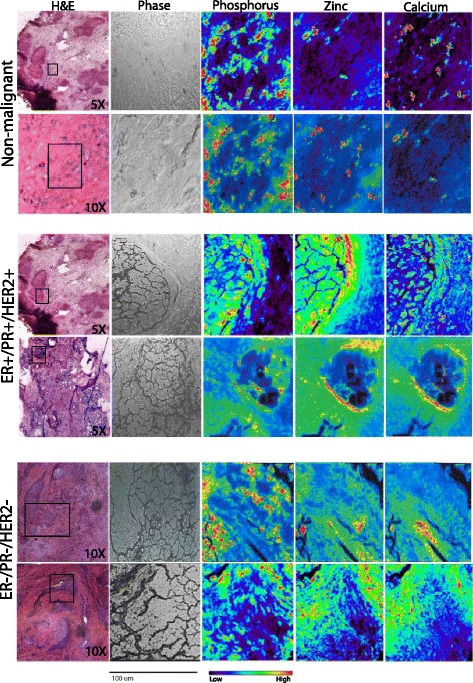
Zinc accumulation reflects breast cancer subtype in human tumors. Representative images of sections (5 μm) of non-malignant breast tissue, Luminal (ER+/PR+/HER2+) or Basal (ER-/PR-/HER2-) tumors (*n* = 2 women/sub/type) stained with hematoxylin and eosin (H & E; 5 or 10× magnification). Malignant regions (noted in box) were visualized by light microscopy (phase; 20× magnification), identified in serial sections (20 μm), and then analyzed by X-ray fluorescence microscopy. The corresponding element (phosphorus, zinc, calcium) is shown above each image. The rainbow color scale reflects the signal intensity measured as micrograms per square centimeter in each pixel, with darker pixels (*purple, blue*) representing areas of low concentration and brighter pixels (*yellow, red*) representing areas of high concentration. A scale bar (100 μm) is shown below the phase contrast images

We first utilized X-ray fluorescence microscopy to determine the spatial distribution of Zn in Luminal and Basal tumors and adjacent normal tissue (Fig. 1). Spatial analysis revealed differences in Zn content and localization within the malignant regions. In Luminal breast tumors, Zn primarily accumulated around the tumor periphery. In Basal breast tumors, Zn was more evenly distributed throughout the malignant tissue. When compared with the distribution of calcium (Ca), some differences were noted such that Zn overlapped closely with Ca in Basal tumors, but this was less consistent in Luminal tumors.

### Zn transporter expression differs between Luminal and Basal tumors

**Fig. 2:**
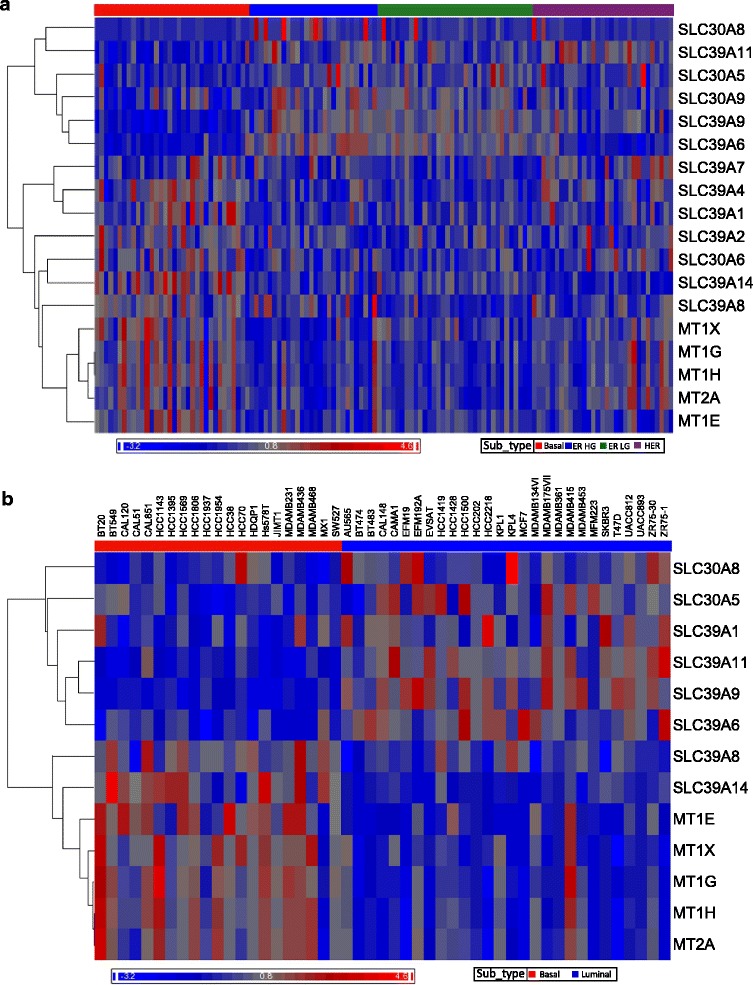
Microarray analysis reveals differential expression of *SLC30A*, *SLC39A*, and *MT* genes in breast cancer subtypes. Subtype dependent differential expression of *SLC30A*, *SLC39A*, and *MT* genes in **a** breast tumors and **b** cell lines were identified using ANOVA and the heat map representing differential expression of significant genes (FDR <0.05) is shown. Breast tumor classification is indicated by the color bar across the top as Basal (*red*), ER^+^-High Grade (ER HG, *blue*), ER^+^-Low Grade (ER LG, *green*), HER (*purple*) and cell lines classified as Luminal (*blue*) and Basal (*red*). The classifications were used as ANOVA factors and the mean log2 expression value was shifted to zero to generate the heatmap

Due to the differences in Zn distribution, we postulated that Basal and Luminal breast tumors would have unique patterns of Zn transporter expression. We analyzed microarray gene expression data derived from human breast tumors and noted a significant effect of subtype on Zn transporter expression (Fig. 2a). Pair-wise comparison of the subtypes show that Basal and ER^+^ tumors had a distinct pattern of *SLC30A*, *SLC39A*, and *MT* gene expression. Significant differences were detected in the expression of *MT1, MT2*, *SLC30A5-6*, *SLC30A8-9*, and *SLC39A1-2*, *SLC39A4*, *SLC39A6-9*, *SLC39A11*, and *SLC39A14* with a false discovery rate (FDR) of *p* < 0.05. In addition, we noted more similarity between the Basal and HER2^+^ subtypes and less similarity between the Basal and ER^+^ subtypes with respect to the expression of the *SLC30A*, *SLC39A*, and *MT* genes (Table 1). Of particular note, we found that *MTs*, *SLC39A4,* and *SLC39A14* were consistently over-expressed while *SLC39A6*, *SLC39A9*, and *SLC39A11* were consistently under-expressed in the Basal subtypes (fold-change >1.5). Finally, among ER^+^ tumors, increasing tumor grade was associated with increased expression of *SLC39A8*. Taken together our data indicated that gene expression of members of the Zn transporting network was substantially different between Basal and Luminal breast tumor subtypes.

**Table 1:** The Zn transporting network is differentially expressed between breast cancer subtypes

	Breast tumor*						Cell line*	Cell line**
	Microarray						Microarray	PCR
Gene^a^	Basal vs ER HG	Basal vs ER LG	Basal vs HER2	ER HG vs ER LG	ER HG vs HER2	ER LG vs HER2	Basal vs Luminal	Basal vs Luminal
MT1E	1.8	1.5	1.8	-	-	-	3.2	Basal only
MT1G	1.5	1.5	-	-	-	-	1.9	
MT1H	1.6	1.6	-	-	-	-	2.2	
MT1×	2.0	1.5	-	-	−1.6	-	2.5	
MT2A	1.3	1.4	-	-	−1.3	−1.4	2.1	
SLC30A1	-	-	-	-	-	1.2	-	-
SLC30A2	-	-	-	-	-	-	-	−3.9
SLC30A4	-	-	-	-	-	-	-	-
SLC30A5	−1.2	−1.2	-	-	-	-	−1.4	−13.6
SLC30A6	1.2	1.2	-	-	-	-	-	−8.5
SLC30A8	−3.1	-	-	-	2.2	-	-	-
SLC30A9	−1.3	−1.3	-	-	-	-	-	5.0
SLC30A10	-	-	-	-	-	-	-	−46.4
SLC39A1	1.3	1.2	-	-	-	-	-	-
SLC39A2	1.2	1.1	-	-	-	-	-	18.0
SLC39A4	1.9	2.2	-	-	-	−1.5	-	−60.0
SLC39A6	−4.1	−3.2	-	-	3.3	2.6	−2.3	-
SLC39A7	-	-	-	-	-	−1.4	-	-
SLC39A8	-	2.3	-	1.9	-	-	2.3	3.0
SLC39A9	−1.8	−1.8	−1.5	-	-	1.2	−1.8	-
SLC39A11	−1.8	−1.6	−2.2	-	-	−1.4	−2.5	−5.7
SLC39A12	1.2	1.2	-	-	-	-	-	-
SLC39A14	1.8	1.4	2.1	-	-	1.4	2.3	-

*ER HG* estrogen receptor positive, high grade, *ER LG* estrogen receptor positive, low grade

*Numbers represent mean fold difference between subtypes analyzed by independent pairwise comparison, *p* < 0.05

**Numbers represent mean fold difference between MDA-MB-231 (basal-like) and T47D (luminal) cells analyzed by Student’s t-test, *p* < 0.05

^a^Only genes that are significantly differentially expressed are indicated

### Zn transporter expression in luminal (T47D) and basal-like (MDA-MB-231) breast cancer cell lines mimics differences in expression in Luminal and Basal tumors

We next analyzed microarray gene expression data of all breast cancer cell lines that were available (Fig. 2b). Statistical analysis of Zn transporter expression indicated that similar to what was observed in breast tumor tissues, basal-like cancer cells also had significantly higher expression of *MT*s and *SLC39A14* and lower expression of *SLC39A6*, *SLC39A9*, and *SLC39A11* (fold-change >1.5; FDR, *p* < 0.05) (Table 1). In sum, genes regulating Zn transport have a subtype-specific pattern of expression in cell lines under normal culture conditions that is similar to that observed in breast tumors, suggesting that malignant cell lines are reliable models for dissecting Zn metabolism and the consequences of dysregulation in breast cancer.

**Fig. 3:**
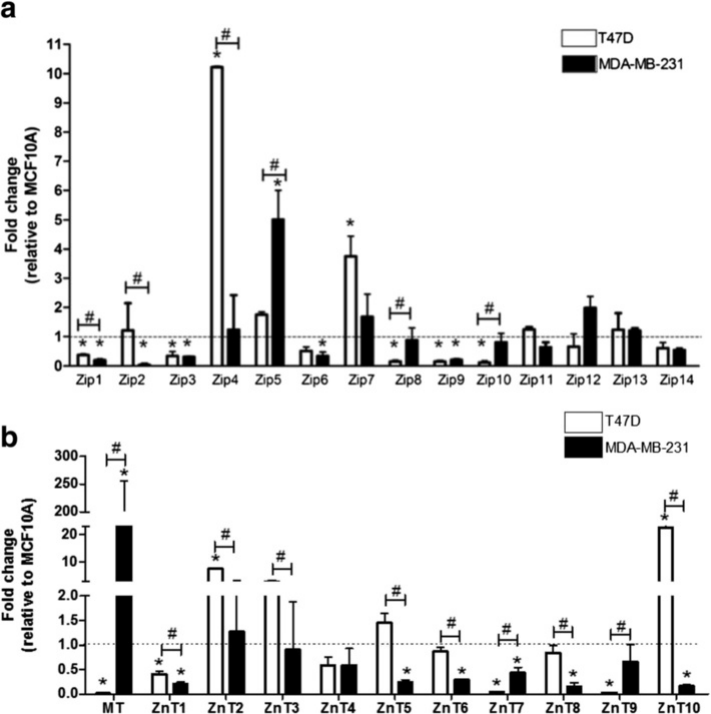
SLC30A, SLC39A, and MT1 genes are differentially expressed in T47D and MDA-MB-231 cells. Expression of members of the SLC39A (ZIP; **a**) or SLC30A (ZnT; **b**) families of Zn transporters in T47D and MDA-MB-231 cells compared with non-malignant MCF10A cells. Data represent mean fold-change relative to non-tumorigenic MCF10A cells (*dashed line*) ± SD, *n* = 4-6. Asterisk indicates a significant difference between malignant cells and MCF10A cells for each gene, *p* < 0.05. Hash-tag indicates a significant difference between subtypes, *p* < 0.05

Based upon these data, we chose T47D and MDA-MB-231 cells as common models representing Luminal and Basal subtypes of breast cancer, respectively. First, we measured the relative gene expression of all Zn transporters and *MTs* using relative real-time PCR (Fig. 3 and Table 1). Similar to our microarray analysis, we identified significant subtype-specific differences in the expression of numerous Zn management proteins. *MT* was only expressed in basal-like MDA-MB-231 cells. Expression of four *SLC30A* (*SLC30A2*, *SLC30A5*, *SLC30A6*, and *SLC30A10*) and two *SLC39A* (*SLC39A4* and *SLC39A11*) genes were significantly lower in basal-like MDA-MB-231 cells compared to luminal T47D cells (*p* < 0.05). Expression of *SLC30A9*, *SLC39A2*, and *SLC39A8* were significantly higher in MDA-MB-231 cells relative to expression in T47D cells (*p* < 0.05).

**Fig. 4:**
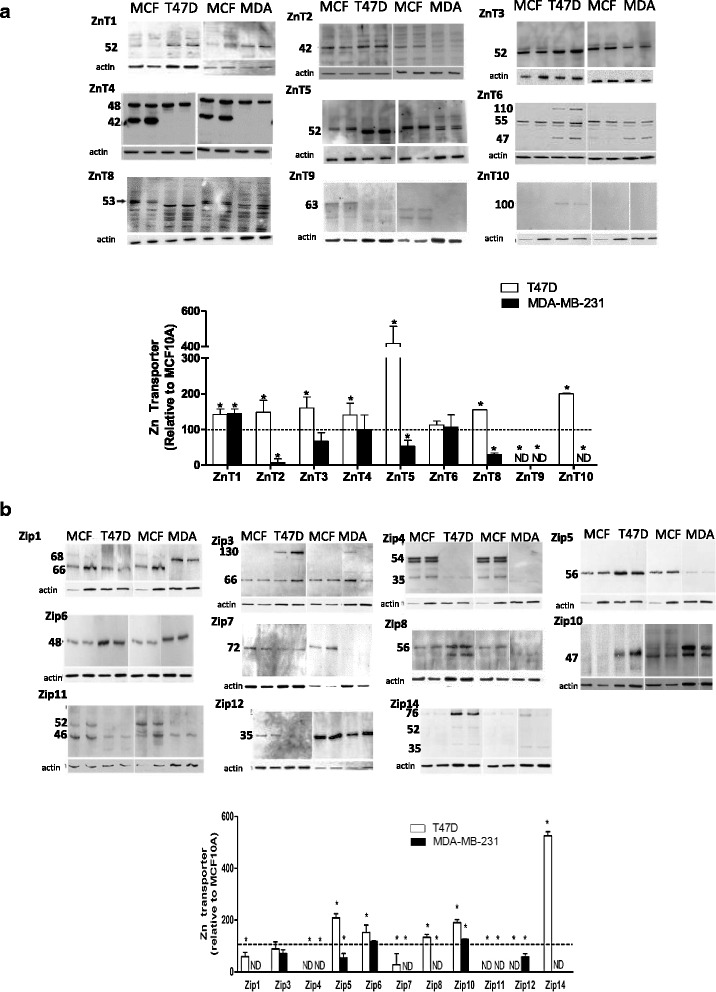
Zinc transporter proteins are differentially expressed in MCF10A, T47D, and MDA-MB-231 cells. **a** Representative immunoblots of ZnT proteins detected in MCF10A, T47D, and MDA-MB-231 cells. Suitable antibodies for ZnT7 were not identified. B-actin was used as a normalization control. Images from different immunoblots or images from different parts of the same immunoblot are separated by dividing lines (for visual consistency). The major isoform was used for quantification. Data represent mean Zn transporter: β-actin ± SD, *n* = 2-3 for each cell line/experiment; each experiment was conducted 2–5 times. Asterisk denotes a significant difference from MCF10A cells, *p < 0.05. ND, not detected. **b** Representative immunoblots of ZIP proteins detected in MCF10A, T47D, and MDA-MB-231 cells. Suitable antibodies for ZIP2 and ZIP9 were not identified. ZIP13 was not expressed in MCF10A, T47D or MDA-MB-231 cells, but was detected in HC11 cells (data not shown). Data represent mean Zn transporter:β-actin ± SD, *n* = 2-3 samples/cell line/experiment and each experiment was conducted 2–5 times. Asterisk denotes a significant difference from MCF10A cells, *p* < 0.05. ND, not detected

We next compared the expression of Zn transporters in T47D and MDA-MB-231 cells to the non-transformed mammary epithelial cell line MCF10A (Fig. 3). *Luminal cells:* Consistent with reports that MT over-expression is associated with invasiveness [23], we detected minimal mRNA expression of *MTs* in non-invasive, T47D cells. T47D cells had significantly lower expression of five *SLC39A* (*SLC39A1*, *SLC39A3*, and *SLC39A8-10)* and three *SLC30A* (*SLC30A1*, *SLC30A7*, and *SLC30A9*) genes relative to expression in MCF10A cells (*p* < 0.05). Expression of two *SLC39A* (*SLC39A4* and *SLC39A7*) and two *SLC30A* (*SLC30A2* and *SLC30A10*) genes were higher relative to expression in MCF10A cells (*p* < 0.05). Next, protein expression profiles were generated for most of the ZIP (*SLC39A* gene family) and ZnT (*SLC30A* gene family) proteins except for ZIP2, ZIP9, and ZnT7, which we were unable to identify suitable antibodies (Fig. 4). Changes in gene expression did not always parallel changes in protein abundance (Table 2). The protein abundance of ZnT9 and several ZIP proteins (ZIP1, ZIP4, ZIP7, and ZIP12) was significantly lower in T47D compared to MCF10A cells, while numerous ZIP (ZIP5, ZIP6, ZIP8, ZIP10, and ZIP14) and ZnT (ZnT1, ZnT2, ZnT3, ZnT4, ZnT5, ZnT8 and ZnT10) proteins were significantly over-expressed in T47D cells compared with MCF10A cells. In addition, the presence of an additional protein much larger than the predicted molecular mass was detected for ZIP3 (~130 kD), ZIP14 (~76 kD), and ZnT6 (~110 kD) in T47D cells. (Only the larger molecular mass of the ZIP14 protein has been reported previously [in U251 cells, Abcam], thus it was included in the quantification). Additionally, ZIP4 and ZIP11 proteins were only robustly detected in MCF10A cells and ZIP13 protein was not detected in human breast cells (data not shown). *Basal-like cells:* Consistent with reports that MT over-expression is associated with invasiveness [23], mRNA expression of *MT*s was ~200-fold higher in basal-like cells relative to expression in MCF10A cells (Fig. 3). MDA-MB-231 cells had lower (>4-fold) expression of six *SLC30A* (*SLC30A1*, *SLC30A5*, *SLC30A6*, *SLC30A7*, *SLC30A8*, and *SLC30A10*) and five *SLC39A* (*SLC39A1*, *SLC39A2*, *SLC39A3*, *SLC39A6*, and *SLC39A9*) genes. Only the expression of *SLC39A5* was significantly higher (~5-fold) relative to its expression in MCF10A cells (*p* < 0.05). Again, changes in gene expression did not parallel changes in protein abundance (Table 2). The protein abundance of four ZnT (ZnT2, ZnT5, ZnT8, and ZnT9) and six ZIP (ZIP4, ZIP5, ZIP7, ZIP8, ZIP11, and ZIP12) proteins was significantly lower, while only two Zn transporters (ZIP10 and ZnT1) were significantly over-expressed at the protein level in MDA-MB-231 compared with MCF10A cells (Fig. 4). A larger isoform (~2 kD larger than expected) of ZIP1 and ZIP10 was detected in MDA-MB-231 cells; however, these isoforms were not detected in MCF10A cells and were not included in the densitometric analysis. ZnT10 and ZIP14 were not detected in MCF10A or MDA-MB-231 cells.

**Table 2:**
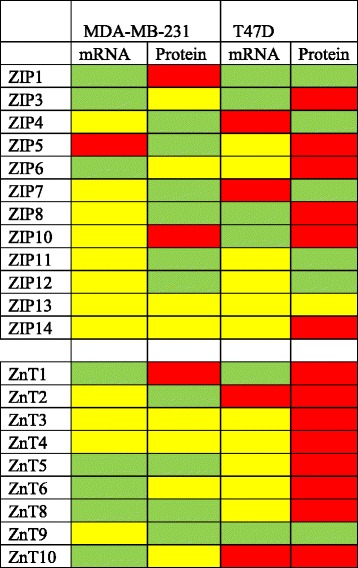
Subtype-specific relationship between changes in mRNA and protein expression relative to non-malignant cells^a^

^a^Green indicates lower expression compared with MCF10A cells; yellow indicates no change in expression compared with MCF10A cells; red indicates higher expression compared with MCF10A cells

### ZnT2 protein is degraded in MDA-MB-231 cells

**Fig. 5:**
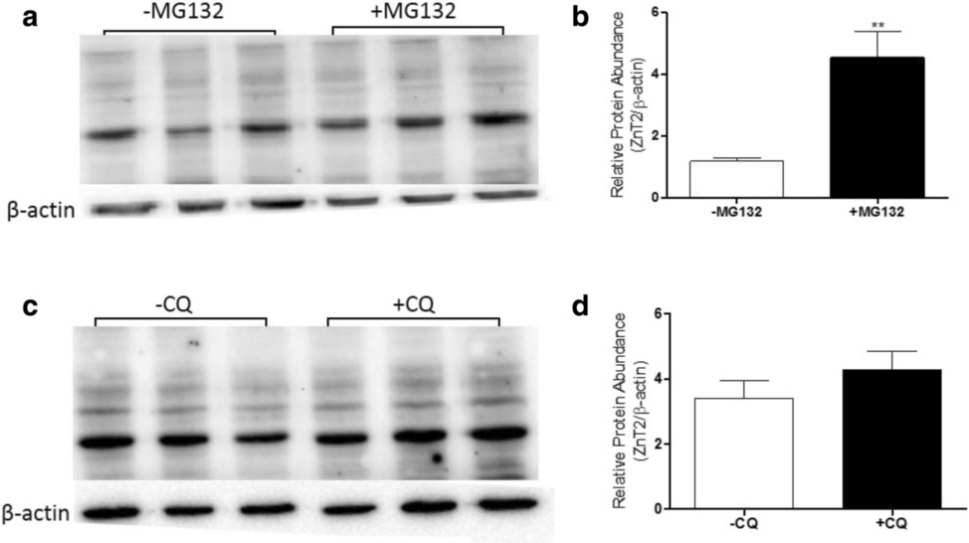
Lack of ZnT2 protein in MDA-MB-231 cells is due to proteasomal degradation. **a** Representative immunoblot of ZnT2 protein detected in MDA-MB-231 cells treated with MG132 (10 μM; proteasome inhibitor) for 6 h. **b** Data represent mean Zn transporter: β-actin ± SD, *n* = 3 samples/cell line. Each experiment was conducted 3 times. Asterisk indicates a significant difference from untreated (−MG132) cells, *p* < 0.05. **c** Representative immunoblot of ZnT2 protein detected in MDA-MB-231 cells treated with chloriquine (CQ, 30 μM; lysosome inhibitor) for 24 h. **d** Data represent mean Zn transporter: β-actin ± SD, *n* = 3 samples/cell line. Each experiment was conducted 3 times

As described above, we noted that while mRNA expression of numerous Zn transporters in MDA-MB-231 cells was similar to/greater than expression in MCF10A cells (ZIP4, ZIP5, ZIP7, ZIP8, ZIP11, ZIP12, ZnT2, and ZnT9), the protein levels were greatly reduced. To provide some mechanistic understanding behind this finding we chose to focus on ZnT2. To determine why ZnT2 protein was minimally detectable in MDA-MB-231 cells, we inhibited proteasomal and lysosomal degradation and measured ZnT2 abundance by immunoblotting. Treatment of MDA-MB-231 cells with the proteasomal inhibitor MG132, but not the lysosomal inhibitor chloroquine, resulted in greater abundance of ZnT2 compared to untreated MDA-MB-231 cells (Fig. 5), suggesting that ZnT2 is proteosomally degraded in MDA-MB-231 cells.

### T47D and MDA-MB-231 cells accumulate more Zn but distribute Zn differently compared to MCF10A cells

**Fig. 6:**
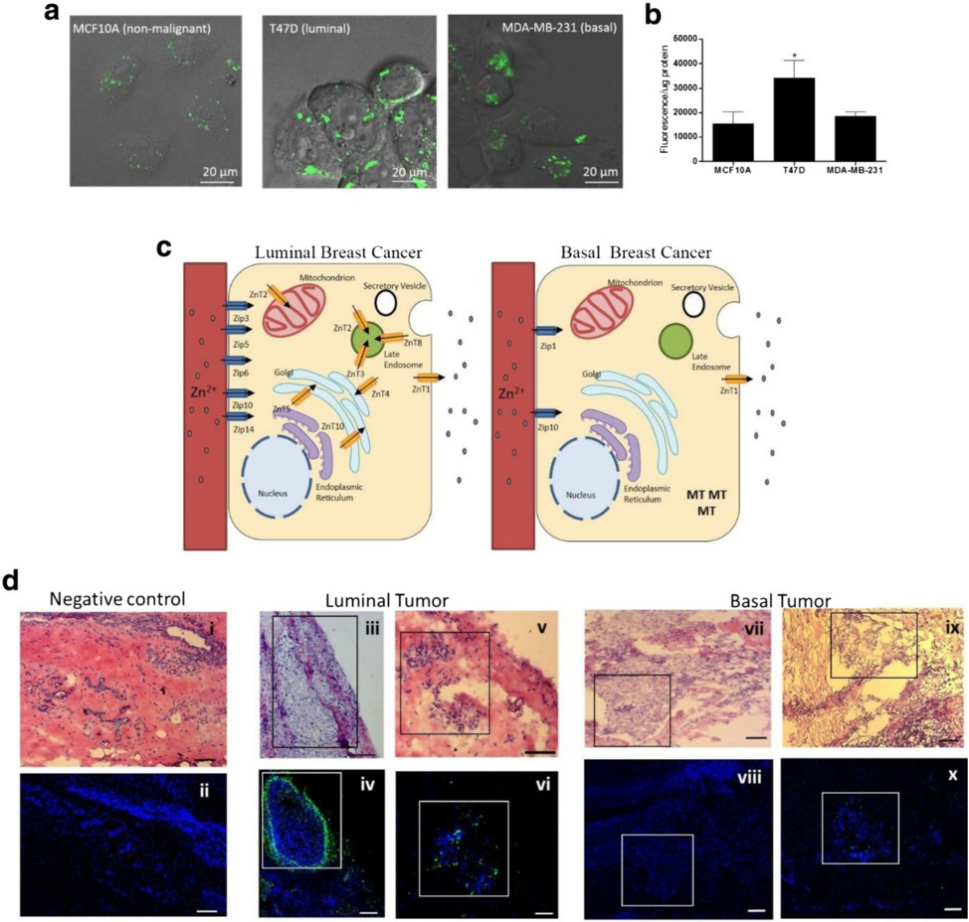
Subcellular Zn accumulation is subtype-specific in breast cancer cells. **a** Confocal micrographs illustrating labile Zn pools (FluoZin-3, *green*) in MCF10A, T47D and MDA-MB-231 cells (60× magnification). Images are presented with differential interference contrast over-lay. Scale bar = 20 μm. **b** Data represent mean FluoZin-3 fluorescence intensity/μg protein ± SEM, *n* = 3 samples/cell line, from 3 independent experiments. Asterisk indicates a significant difference from MCF10A cells, *p* < 0.05. **c** Cartoon depicting Zn transporters that are over-expressed (>1.4-fold) and subcellular Zn pools that are increased in T47D cells (a model of Luminal breast cancer) and MDA-MB-231 cells (a model of Basal breast cancer) compared to non-tumorigenic MCF10A cells. **d** Representative images (5× magnification) of sections (5 μm) of non-malignant breast tissue and human tumors from two different women stained with hematoxylin and eosin (H&E; *iii, v, vii, ix*) and visualized by light microscopy (10× magnification); serial sections were immunostained for ZnT2 (*iv*, *vi*, *viii*, *x*). Negative controls were treated similarly but incubated with normal rabbit IgG. Malignant regions are noted in boxes in both H&E and immunofluorescent images. Scale bar =100 μm)

We next assessed differences in Zn content and distribution within these breast cancer and non-malignant cells. We first quantified the total amount of Zn in cell lysates using atomic absorption spectroscopy. Similar to observations in breast tumors, T47D cells accumulated ~5-fold greater Zn (0.276 ± 0.01 μg Zn/mg protein) while MDA-MB-231 cells accumulated ~2.5-fold greater Zn (0.107 ± 0.02 μg Zn/mg protein) relative to MCF10A cells (0.057 ± 0.06 μg Zn/mg protein), *p* < 0.05. To visualize the localization of Zn pools within these distinct subtypes, we used the Zn-responsive fluorophore FluoZin-3 [23] which fluoresces upon Zn binding (kD = 4–15 nM) [24]. We noted that the staining pattern of FluoZin-3 was distinctly different in each cell line (Fig. 6a). Labile Zn pools in MCF10A cells were confined to small peri-nuclear vesicles, consistent with our previous observation that Zn accumulates in the Golgi apparatus/endoplasmic reticulum in normal mammary cells [25]. In contrast, labile Zn pools in T47D cells were enriched in numerous large punctate intracellular vesicles, while in MDA-MB-231 cells, hazy fluorescence was noted throughout the cytoplasm and in a large, amorphous subcellular compartment(s). Importantly, consistent with the measurements of total Zn concentration noted above, quantification of FluoZin-3 fluorescence suggested that T47D cells accumulated significantly greater labile Zn compared with MCF10A and MDA-MB-231 cells (Fig. 6b). Collectively, Zn transporter protein profiling (setting a threshold of a >1.4-fold change in protein abundance) combined with the visualization of sub-cellular Zn pools using FluoZin-3, allowed us to generate a model of the Zn ionome (the profile of subcellular Zn transporters and Zn distribution) in luminal and basal-like breast tumor cells (Fig. 6c). This modeling illustrates that increased abundance of numerous ZIP and vesicular ZnT proteins (ZnT2, ZnT3, ZnT4, ZnT5, ZnT8, and ZnT10) is associated with enhanced vesicular Zn accumulation in luminal cells, while increased expression of only two ZIP proteins and MT is associated with more moderate Zn accumulation in basal-like cells.

### ZnT2 staining is greater in Luminal breast tumors compared with Basal tumors and adjacent non-malignant tissue

Because ZnT2 expression is positively associated with vesicular Zn accumulation in malignant breast cancer cells, we next used immunofluorescent imaging in Luminal and Basal tumors to determine if the abundance of ZnT2 was similar to our observations in luminal and basal-like breast cancer cells. Indeed, we found that ZnT2 was primarily detected in Luminal tumors and in some cases was enhanced around the periphery (Fig. 6d), similar to the pattern of Zn accumulation observed using Xray fluorescence microscopy (Fig. 1). However, this peripheral staining pattern in Luminal tumors was not consistently observed. Moreover, minimal ZnT2 was detected in Basal tumors, collectively reflecting the results obtained in cultured breast cancer cells.

### The loss of ZnT2 in MDA-MB-231 cells contributes to the invasive malignant phenotype

**Fig. 7:**
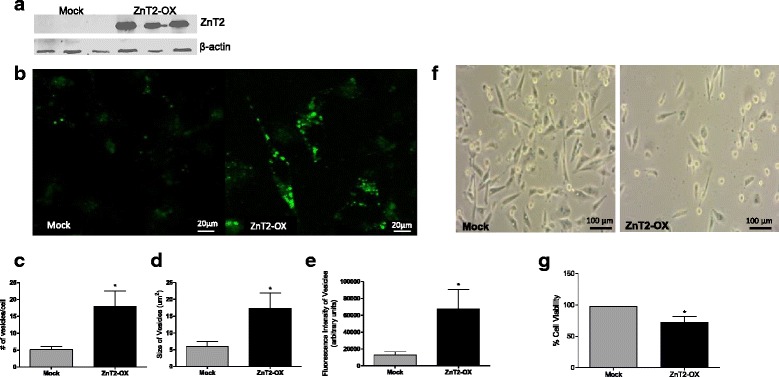
ZnT2 over-expression in MDA-MB-231 cells caused vesicular Zn sequestration and decreased cell viability. **a** Representative immunoblot of ZnT2-HA in ZnT2-over-expressing (ZnT2-OX) MDA-MB-231 cells. **b** Representative confocal images of FluoZin-3 fluorescence (*green*) in Mock-transfected and ZnT2-OX MDA-MB-231 cells. 60× magnification. Scale bar = 20 μm. **c** Data represent mean number of vesicles ± SEM, *n* = 8 cells/field from 3 different fields. Asterisk indicates a significant difference from Mock transfected controls, *p* < 0.05. **d** Data represent mean size of vesicles (μm) ± SEM, *n* = 8 cells/field from 3 different fields. Asterisk indicates a significant difference from Mock transfected controls, *p* < 0.05. **e** Data represent mean fluorescence intensity/cell ± SEM, *n* = 8 cells/field from 3 different fields. Asterisk indicates a significant difference from Mock transfected controls, *p* < 0.05. **f** Representative phase contrast images of Mock-transfected and ZnT2-OX MDA-MB-231 cells. 10× magnification. Scale bar = 100 μm. **g** Cell viability was assessed using the trypan blue exclusion assay. Data represent mean number of viable cells ± SD, *n* = 3 samples/genotype, from 3 independent experiments. Asterisk indicates a significant difference from Mock transfected controls, *p* < 0.05

To test the hypothesis that the ability to sequester Zn in vesicles via ZnT2 is an important driver of the molecular phenotype, we expressed ZnT2 in MDA-MB-231 cells and assessed the functional response (Fig. 7). We chose this approach because ZnT2 is a vesicular transporter important for Zn accumulation [26] and protection against Zn cytotoxicity [27], this approach complemented our previous report that ZnT2 attenuation in T47D cells reduces tumorigenesis [18], and ZnT2 was significantly over-expressed in luminal cells and Luminal tumors and not expressed in basal-like cells and Basal tumors. In the current set of studies, we found that ZnT2 over-expression in MDA-MB-231 cells (Fig. 7a) led to Zn vesicularization and decreased cell viability. FluoZin-3 imaging revealed that in MDA-MB-231 cells transfected to express ZnT2, labile Zn accumulated in vesicles throughout the cell (Fig. 7b). Moreover, there were significantly more vesicles (Fig. 7c) and vesicles of a larger size (Fig. 7d) compared to vesicles in the mock transfected cells. In addition, the overall fluorescence intensity of vesicles per cell (Fig. 7e) was significantly greater suggesting that ZnT2 over-expressing cells accumulated more vesicular Zn. This was associated with a concurrent change in morphology (Fig. 7f) and a ~25 % reduction in cell viability (Fig. 7g).

**Fig. 8:**
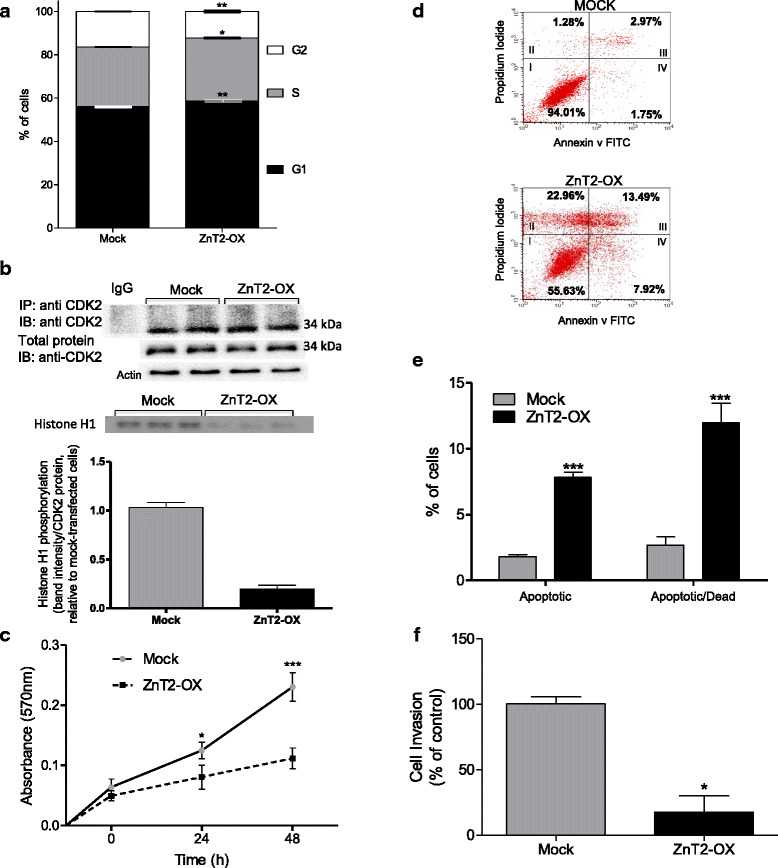
ZnT2 over-expression in MDA-MB-231 cells causes alterations in cell cycle, increased apoptosis and decreased invasion. **a** Cell cycle analysis was measured using in Mock-transfected and ZnT2 over-expressing (ZnT2-OX) MDA-MB-231 cells by flow cytometry. Data represent mean % of cells ± SEM, *n* = 3 samples/genotype from 3 independent experiments. Asterisk(s) indicates a significant difference from Mock transfected controls, *p < 0.05; **p < 0.01. **b** Cyclin dependent kinase 2 (CDK2) kinase activity in MDA-MB-231 cells. (*Top panel*) Representative immunoblot of immunoprecipitated CDK2, total CDK2 and β-actin in Mock-transfected and ZnT2-OX MDA-MB-231 cells. (*Bottom panel*) CDK2 kinase activity was assessed using histone H1 as a substrate. Representative gel illustrating ^32^P labeling of histone H1 and corresponding relative densitometric analysis. Data represent mean band intensity (normalized to CDK2 protein) ± SD, *n* = 3 samples/genotype, from 2 independent experiments. Asterisks indicates a significant difference from Mock transfected controls, ***p < 0.001. **c** Cell proliferation in Mock-transfected and ZnT2-OX MDA-MB-231 cells. Data represent mean absorbance ± SEM, *n* = 3 samples/genotype, from 3 independent experiments. Asterisk(s) indicates a significant difference from Mock-transfected controls, *p < 0.05; **p < 0.01. **d** Representative forward-by-side scatter plot depicts the population of early apoptotic (*I*), apoptotic (*IV*), apoptotic/dead (*III*) and dead cells (*II*). **e** The percentage of apoptotic and dead cells was significantly higher in ZnT2-OX cells compared to Mock-transfected cells. Data represent mean % cells ± SEM *n* = 3 samples/genotype from 2 independent experiments. Asterisks indicates a significant difference from Mock-transfected controls, ***p < 0.001. **f** ZnT2-OX resulted in a significant reduction in invasion (~80 %) compared with Mock-transfected controls. Data represent mean % control ± SEM *n* = 3 samples/genotype from 2 independent experiments. Asterisk indicates a significant difference from Mock-transfected controls, *p < 0.05

To better understand the effects of ZnT2 over-expression on cell viability, we analyzed the effects of ZnT2 over-expression on cell cycle and CDK2 activity. CDK2 is a cell cycle protein that is critical for the G1/S transition. We observed distinct shifts in cell cycle in ZnT2 over-expressing cells compared to mock-transfected cells (Fig. 8a). Immunoblot analysis and kinase activity assays for CDK2 revealed that Zn sequestration in ZnT2 over-expressing cells had no effect on CDK protein abundance (Fig. 8b; top panel); however, CDK2 activity was significantly reduced by ~80 % (Fig. 8b; bottom panel). Shifts in cell cycle in ZnT2 over-expressing cells were also associated with decreased cell proliferation (Fig. 8c) and increased apoptotic cell death (Fig. 8d and e). However, most importantly, the molecular phenotype was profoundly affected, as invasion was significantly reduced (80 %) in ZnT2 over-expressing MDA-MB-231 cells (Fig. 8f).

## Discussion

Zn hyper-accumulates in breast tumors [4] and breast cancer cells [18]; however, the relevance of Zn accumulation and the relationship to the molecular phenotype is not understood. Consistent with previous reports, we found that not only does Zn accumulate, but that Zn distribution and the entire Zn transporting network was profoundly different. Importantly, our study revealed functionally relevant subtype-specific differences in Zn dysregulation between Luminal and Basal breast tumors and luminal and basal-like breast cancer cells, and provides direct evidence that the ability to sequester Zn into a vesicular compartment underlies the malignant phenotype.

Zn is a critical regulator of multiple cellular processes including DNA transcription, cell signaling, proliferation, invasion, apoptosis, and autophagy [7], thus Zn mismanagement may underlie hallmarks such as malignant transformation, tumorigenesis, invasion, and metastasis. Zn accumulation within the Golgi/vesicular compartment may be of particular functional relevance. The Golgi/vesicular compartment contains many apoptotic regulatory components such as Fas, Hippi protein, tumor necrosis factor receptor-1, Bcl-2 family members, and caspase-2 [28]. Fas is activated by Zn depletion in hippocampal neurons [29] and conversely, Zn decreases abundance of NFκB and Bcl-2 family members [30]. Thus Zn accumulation or depletion in the Golgi/vesicular compartment may profoundly affect cell function. In addition, the Golgi apparatus provides a platform for RAS/MAPK [31], RANK/NFκB [32] and PI3K [33] signaling. The role of Zn in cell signaling is multifactorial and complex and involves (in)activation of phosphorylation pathways, modulation of cAMP and cGMP activity via degradation by Zn-dependent cyclic nucleotide phosphodiesterases, and perhaps direct binding of Zn to TRAF6, the upstream effector of MEK and NFkB activation [34]. Intriguingly, ZIP7, ZIP11 and ZIP13 are reported to localize to the Golgi apparatus or intracellular vesicles [35, 36]. Moreover, ZIP7 [37], ZIP13 [38], and ZIP14 [39] have been shown to specifically activate EGFR, TGFβ, and G-protein coupled receptor-mediated cAMP-CREB signaling, respectively. Thus the loss of ZIP7, ZIP11, and ZIP13 expression that we noted in both T47D and MDA-MB-231 cells suggests that aberrant expression and/or function of these Zn transporters in particular, may underlie defective cell signaling in malignant breast cells.

Previous studies have found that over-expression of MT [21, 23, 40], ZIP6 [14, 15, 41], ZIP10 [13], and ZnT2 [18] is associated with breast cancer. Increased MT expression has been associated with chemoresistance [21–23] and is correlated with increased matrix metalloproteinase expression, a Zn-dependent enzyme important for the degradation of extracellular matrix and thus invasion/metastasis. ZIP6 expression is regulated by estrogen, is upregulated in ER^+^ breast tumors, and has been associated with chemotherapy resistance [14, 15, 41]. ZIP10 expression is associated with increased motility and invasiveness in triple-negative breast cell lines [13], and ZnT2 is over-expressed in luminal breast cancer cells [18]. Our data now dramatically expands this current list of Zn transporters that are dysregulated in breast cancer, as we found that increased abundance of ZIP10 and loss of ZIP4, ZIP7, ZIP11, and ZnT9 were universal mechanisms associated with Zn hyper-accumulation in malignant breast cells. Moreover, a key finding from our study was that dysregulation in the Zn transporting network was extensive and subtype-specific. While numerous Zn transporters were over-expressed in luminal cells (ZIP3, ZIP5, ZIP6, ZIP8, ZIP10, ZIP14, ZnT1, ZnT2, ZnT3, ZnT4, ZnT5, ZnT8, and ZnT10), only a few were over-expressed in basal-like cells (MT, ZIP10 and ZnT1). Using FluoZin-3 as a Zn reporter, we found that changes in Zn transporters corresponded to subtype-specific alterations in sub-cellular Zn pools. While MDA-MB-231 cells hyper-accumulate a modest amount of Zn, the excess Zn likely exists primarily bound to MTs to protect cells from Zn cytotoxicity [8, 27]. In contrast, T47D cells accumulate much greater Zn than MDA-MB-231 cells and Zn is sequestered in the vesicular compartment, consistent with the over-expression of the Golgi/vesicular Zn transporters ZnT2, ZnT3, ZnT4, ZnT5, ZnT8, and ZnT10 [25, 42–45]. While further studies using novel genetically encoded Zn reporters [46] are required to better understand the identity and function of these intracellular Zn pools, these data strongly implicate differences in Zn distribution as a defining characteristic of the molecular subtype of breast cancer.

We previously reported that the inability to accumulate Zn in vesicles, by attenuating ZnT2 in T47D cells, results in cytoplasmic Zn accumulation, oxidative stress and autophagic cell death [18]. Here, we extend those observations and report that Zn accumulation into the vesicular compartment by expressing ZnT2 in MDA-MB-231 cells resulted in shifts in cell cycle as a result of decreased CDK2 kinase activity. CDK2 is required for the G1/S phase transition in the cell cycle. The vesicularization of Zn concomitant with decreased CDK2 activity suggests that CDK2 activity is a Zn-dependent process. To our knowledge, this is the first report of the Zn-dependency of CDK2. Decreased CDK2 activity ultimately led to reduced proliferation and increased apoptosis consistent with recent studies that have shown that CDK2 inhibition is required for apoptosis [47–49]. However, the most provocative finding was that ZnT2 over-expression and Zn vesicularization caused an 80 % reduction in invasion. This provides further evidence that modulation of sub-cellular Zn pools in malignant breast cells plays an important role in the regulation of cell phenotype and suggests that the inability to vesicularize Zn pools may reflect and/or promote the aggressiveness of breast cancer; however, additional studies are needed to understand the relevance of Zn accumulation into vesicles on these critical phenotypic hallmarks in breast cancer.

Our study also highlights the need to move beyond the analysis of mRNA expression when interpreting functional relationships between Zn transporters and disease. As previously reported [50], gene and protein expression are not always positively correlated, which reaffirms that post-transcriptional regulatory mechanisms including miRNA binding [51], mRNA stability and protein cleavage [52], and protein ubiquitination [53] are critical determinants of Zn transporter regulation (see Additional file 1: Table S1). Here, our data suggest that post-transcriptional regulatory mechanisms may be subtype-specific for ZIP1, ZIP3, ZIP10 and ZIP14, as numerous isoforms were differentially expressed in specific breast cancer sub-types. Moreover, our observations regarding ZnT2 in breast cancer cells directly illustrate this post-transcriptional regulation. In contrast to observations in T47D cells, we found that while ZnT2 mRNA expression in MDA-MB-231 cells was similar to that in MCF10A cells, there was significantly less ZnT2 protein in MDA-MB-231 cells. Similar to estrogen receptor alpha [54], we found that ZnT2 was robustly degraded in the proteome, perhaps as a feature of enhanced proteasomal degradation machinery [55]. However, another postulate is that ZnT2 may also be post-transcriptionally regulated by microRNAs. In fact, 16 miRNAs are predicted to bind to ZnT2 mRNA (http://www.microrna.org/microrna/getMrna.do?gene = 7780&utr = 22874&organism = 9606). Of these 16 miRNAs, 2 miRNAs (miR-24 and miR-96) are associated with breast cancer (http://mircancer.ecu.edu) and importantly, 3 miRNAs (miR-24, miR-30a and miR-149) are upregulated in MDA-MB-231 cells [56, 57]. Further studies are required to understand the differential post-transcriptional regulation of ZnT2 in breast cancer subtypes.

## Conclusion

Targeted profiling of breast tumors and malignant cell lines has identified the Zn transporting network as a key centroid of subtype-specific dysregulation. Manipulation of the Zn transporting network and sub-cellular Zn pools decreased malignancy in aggressive, triple-negative breast cancer cells. These data suggest that defects in the subcellular Zn microenvironment may play a key role in alterations in apoptosis, oxidative stress and/or cell signaling, which influences the behavior of malignant breast cancer cells. This may be critical in understanding the molecular differences in phenotype between Luminal and Basal subtypes and potentially elucidate novel diagnostic or therapeutic avenues.

## Methods

### Microarray analysis

A breast cancer cell line dataset (GSE12777, [58]) and a breast tumor dataset (GSE5460, [59]) was used in the analysis. The analysis was performed using the PARTEK software [60]. The cel files were normalized using RMA and the batch effect was corrected using scan date and subtype as ANOVA factors within PARTEK batch-correction implementation. Finally, each array was scaled to the grand median to ensure accuracy of inter-array comparisons. Breast tumors were classified into Basal, ER-High Grade (ER HG), ER-Low Grade (ER LG) and HER [61]. Breast cancer cell lines were classified into basal and luminal based on previously published work and the unassigned cell lines were classified based on cluster membership after Hierarchical clustering of the top 10 % highly variant genes [62, 63]. The Jetset definitions for “best-probeset” (jetset.scores.hgu133plus 2_0.99.3.csv) available for download at [64] was used to restrict the analysis to one probe-set per gene [39]. Subsequent analysis was performed on the *SLC30A1-A10* (ZnT proteins), *SLC39A1-A14* (ZIP proteins) and the metallothionein genes *MT1E*, *MT1F*, *MT1G*, *MT1H* and *MT2A*. The latest Affymetrix annotation (June 2011) assigns probeset 219215_s_at to *SLC39A4* and 202667_s_at to *SLC39A4* and *SLC39A7*. However both Jetset and GeneAnnot [65] assigned probeset 202667_s_at to *SLC39A7* and 219215_s_at to *SLC39A4*, therefore we followed the Jetset/GeneAnnot definitions and retained both probesets in our analysis. ANOVA was used to identify differentially expressed genes and the FDR (step-up) corrected *p*-value <0.05 was used as the cut-off criteria for selecting significant genes. Hierarchical clustering based on Spearman rank dissimilarity of gene expression values and complete linkage was used to generate the heatmaps.

### Cell culture

Human malignant luminal ER^+^/PR^+^/HER2^−^ (T47D), basal-like ER^−^/PR^−^/HER2^−^ (MDA-MB-231) and non-malignant (MCF10A) breast cells were chosen to represent three different breast cell subtypes. Cells were obtained from the American Type Culture Collection (ATCC, Manassas, VA). T47D cells were maintained in growth medium containing, RPMI 1640 (SIGMA, St. Louis, MA) supplemented with fetal bovine serum (10 %), insulin (0.2 units/mL), sodium pyruvate (1.0 mM) and penicillin/streptomycin (1 %). MDA-MB-231 cells were maintained in L15 medium containing penicillin/streptomycin (1 %) and horse serum (10 %). MCF10A cells were maintained in 171 Medium supplemented with Mammary Epithelial Growth Supplement (Invitrogen, Carlsbad, CA). All culture mediums contained ~5 μM Zn as assessed by atomic absorption spectroscopy. Cells were routinely cultured in plastic 75 cm^2^ flasks and sub-cultured every 4–5 days. Cells were maintained in a humidified chamber in 5 % CO_2_ at 37 °C.

### Cellular zinc concentration

Cells were cultured on 15 cm^2^ polycarbonate dishes in growth medium until 90–100 % confluent. Cells were initially rinsed with PBS, and then rinsed with PBS plus EDTA (1 mM) to remove any loosely bound Zn. Cells were collected by gentle scraping and pelleted by centrifugation at 2000 g for 10 min at 4 °C. Cellular protein concentration was determined by the Bradford assay. Cells were resuspended in Ultrex II Nitric Acid (0.5 mL, VWR, West Chester, PA) in mineral-free polypropylene vials and digested at room temperature overnight. Zn concentration was analyzed by atomic absorption spectroscopy using an Atomic Absorption Analyst 400 (Perkin Elmer, Waltham, MA) with WinLab32 software. Data was normalized to total protein content measured by the Bradford assay.

### Imaging and quantification of cellular zinc pools

Labile Zn pools were characterized and visualized as previously described [25]. Cells were seeded onto glass coverslips and cultured overnight until 60–90 % confluent. Cells were rinsed twice with PBS then loaded with FluoZin-™3 AM (1 μM in DMSO containing pluronic acid 127 to a final concentration of 0.02 %; Invitrogen, USA) following manufacturer’s instructions in Opti-MEM for 1 h at 37 °C. Cells were briefly rinsed twice with PBS and washed with PBS for 30 min at 25 °C with constant shaking. Images were collected from live cells using a FV-1000 confocal microscope (Penn State Microscopy and Cytometry Facility). Number, size and the fluorescence intensity of vesicles were analyzed using Imaris® software (Connecticut, USA).

### X-ray fluorescence microscopy

Discarded human tissue samples were collected under Dana-Farber Harvard Cancer Center institutional review board protocol #93-085. Tissue samples were plunge-frozen in ice-cold isopentane bath and processed for microscopy as previously described [25]. Frozen, unfixed tumors were sectioned (5 μm), mounted on positively-charged glass slides, dried at room temperature, post-fixed in phosphate-buffered 4 % paraformaldehyde, washed three times with 1× PBS at 4 °C for 5 min and stained with hematoxylin and eosin (H & E). Briefly, sections were air dried for 4 min, stained with 0.1 % hematoxylin for 2 min and rinsed in ddH_2_O for 5 min. Sections were then stained with 0.5 % Eosin times and then rinsed in distilled H_2_O 3 times. Sections were then dehydrated in ethanol and rinsed in xylenes. Regions of malignant tissue were identified. Serial sections (20 μm) were used for X-ray fluorescence microscopy and imaged with the scanning x-ray microprobe at beamline 2-ID-E at the Advanced Photon Source (Argonne, IL), quantified and processed as previously described [25].

### Immunofluorescence microscopy

Frozen, unfixed tumors were sectioned (5 μm) with a Microm HM 505 E cryostat (GMI, Ramsey, MN) at −25 °C and briefly dried at room temperature onto positively-charged slides. Sections were then post-fixed in phosphate-buffered 4 % paraformaldehyde at room temperature for 15 min. Following fixation, sections were washed with 1× PBS at 4 °C for 5 min; this was repeated 3 times. Sections were treated with 3 % H_2_O_2_ for 10 min and washed twice in 1× PBS for 5 min. Sections were then permeabilized with 0.2 % triton X-100 in 1× PBS for 45 min at room temperature. Sections were blocked (0.1 % heat inactivated goat serum, 1 % BSA, 0.3 % Triton X-100 in 1× PBS) for 1 h at room temperature in a humidified chamber. ZnT2 antibody [66] was diluted in blocking buffer (4 μg/mL) and sections were incubated overnight at 4 °C in a humidified chamber. Sections were washed 3 times with 1× PBS for 5 min and then incubated with DAPI (175 μg/mL) for 10 min at RT, then washed three times with 1× PBS for 5 min. Tissue was mounted in ProLong® Diamond Antifade (ThermoFisher Scientific, USA) and the coverslips were sealed with nail polish.

### RNA analysis

Total RNA was isolated from cells using Trizol (Invitrogen, Carlsbad, CA) according to manufacturer’s instructions. RNA was quantified by spectrophotometry and the integrity was assessed by examination of 28S and 18S bands in 2 % agarose gel electrophoresis. cDNA was synthesized from 1.0 μg of total RNA using TaqMan® reverse transcription kit (Applied Biosystems, Foster City, CA) in 25 μl reaction mixture following manufacturer’s instructions. The reaction mixture was incubated at 25 °C for 10 min, then at 48 °C for 30 min and heated to 95 °C for 5 min. cDNA products were stored at −20 °C until used for semi-quantitative PCR. Semi-quantitative PCR was performed using the DNA Engine Opticon 2 System real-time thermocycler (BioRad, Hercules, CA) coupled with SYBR Green technology (BioRad) and gene-specific primers to human Zn transporters (*SLC39A1-14* and *SLC30A1-10*), MT (predicted to detect *MT1A*, *MT1C*, *MT1D*, *MT1E*, *MT1F*, *MT1H*, *MT1L*, *MT1S*, *MT1X* and *MT2A*) and human β-actin (Primer 3 Input v4.0). The PCR cycling parameters were as follows: 95 °C for 10 min, and 40 cycles of 95 °C for 15 s, 60 °C for 30 s and 72 °C for 30 s. The linearity of the dissociation curve was analyzed by Opticon 2 System software and the mean cycle time of the linear part of the curve was designated *C*t. Each sample was analyzed in duplicate and normalized to β-actin using the following equation: Δ*C*t_gene_ = *C*t_gene_ − *C*t_β-actin_. The difference in expression between MDA-MB-231 and T47D cells was calculated using the following equation: 2^(ΔΔ*C*t)^, (ΔΔ*C*t = mean ΔCt_gene_ in MDA-MB-231 cells − mean ΔCt_gene_ in T47D cells. Values represent mean fold change ± SD, relative to MDA-MB-231 cells (set to 100 %). The difference in expression between MDA-MB-231 or T47D cells and non-malignant MCF10A cells was calculated using the following equation: 2^(ΔΔ*C*t)^, (ΔΔ*C*t = mean ΔCt_gene_ in MCF10A cells − mean ΔCt_gene_ in MDA-MB-231 or T47D cells. Values represent mean fold change ± SD, relative to MCF10A cells (set to 100 %).

### Immunoblotting

Total membrane proteins were isolated from cultured cells, electrophoresed (20–100 μg/sample) and transferred to nitrocellulose as previously described [66]. Additional file 1: Table S1 identifies the antibodies used and the molecular mass of Zn transporters in various cell lines and tissues that have been reported in the literature. Antibodies used in this study are noted. Membranes were blocked for 1 h in 5 % non-fat milk in PBS/0.1 % Tween-20 (PBS-T) and washed 3 times in PBS-T, followed by incubation with antibodies directed against Zn transporters for 45 min then washed 3 times in PBS-T. We were unable to identify suitable antibodies for ZnT7, ZIP2 and ZIP9. Proteins were detected following incubation with donkey, anti-rabbit IgG (1:30,000) conjugated to horseradish peroxidase (Amersham Pharmacia Biotech), visualized with Super Signal Femto Chemiluminescent Detection System (Pierce, Rockford, IL) and exposed to autoradiography film. Membranes were stripped and reprobed for β-actin to control for equal protein loading. Relative band density was quantified using the Carestream Gel Logic 212 Pro and the ratio of Zn transporter: β-actin was used for analysis. Samples were run in duplicate or triplicate and immunoblots were repeated 3–5 times. ZnT2 over-expression in transfected cells was confirmed by incubating membranes with anti-HA antibody (0.8 μg/mL) for 1 h, detected with secondary antibody labeled with IRDye (1:20,000) for 1 h protected from light. ZnT2-HA was detected using the LI-COR® Odyssey CLx System (LI-COR; Lincoln, NE).

### Proteasomal and lysosomal inhibition

MDA-MB-231 cells were plated in 6-well plates at a cell density of 5 ×10^5^. Twenty-four h later, MDA-MB-231 cells were treated with either 30 μM of MG132 (Sigma-Aldrich) for 6 h or 10 μM of Chloroquine Diphosphate salt (MP Biomedicals, LLC; Solon, OH) for 24 h. Total membrane preps and immunoblot analysis were executed as described above. Briefly, ZnT2 was detected following incubation with anti-rabbit IgG (1 μg/mL) conjugated to horseradish peroxidase and visualized with Super Signal Femto Chemiluminescent Detection System (Pierce, Rockford, IL) on a FluorChem System (ProteinSimple, San Jose, CA). Nitrocellulose membranes were stripped once and reprobed for β-actin. Relative densitometry was quantified using the AlphaView® Software (ProteinSimple, San Jose, California). ZnT2 densitometry was normalized to β-actin and used for analysis. Samples were run in triplicate and immunoblots were repeated 2–3 times.

### Transfection

ZnT2 over-expression in MDA-MB-231 cells utilized the Lipofectamine® 2000 Transfection Reagent delivery system (Thermo Scientific, Grand Island, NY). MDA-MB-231 cells were plated at a cell density of ~5 × 10^5^ in 6-well plates (Corning®, USA) and cultured until ~80–90 % confluence. Cells were transfected with Lipofectamine® 2000 and plasmid containing ZnT2-HA [26] (2.5 μg) at a ratio of 2.8:1 (transfection reagent: DNA ratio) in Opti-MEM® (Life Technologies, USA) per manufacturer’s instructions. Cells were transfected for 5 h, after which the transfection medium was removed and replaced with antibiotic-free, normal growth medium. All experiments were done 24 h post transfection. Transfection was confirmed by immunoblotting as described above.

### Proliferation assay

Proliferation was determined with the MTT Cell Growth Determination Kit (Sigma-Aldrich, USA). MDA-MB-231 cells were transfected as described previously in a 6-well plate. Cells were trypsinized and plated in four 96-well plates at a cell density of 5 × 10^3^. Cells were allowed to grow for up to 48 h. Time 0 represents cells plated and analyzed on the same day (~6 h after cells attach). Cells were treated with 3-[4, 5-dimethylthiazol-2-yl]-2, 5-diphenyl tetrazolium bromide (MTT) as instructed by the manufacturer. Briefly, 10 μL of MTT was added aseptically per well and incubated for 3 h. Medium was removed and 100 μL of MTT solvent added. Absorbance was read at 570 nm. Data represent mean ± SD, *n* = 3 samples/genotype from 3 independent experiments.

### Cell cycle analysis

Cells were plated (3 × 10^5^) in a 6-well plate and transfected as described previously. Cells were collected 24 h post-transfection and suspended in cold 70 % ethanol and stored at −20 °C for 24 h. Cells were stained with propidium iodide (BD Biosciences; San Jose, CA) and analyzed by flow cytometry using a FACSCALIBUR (BD Biosciences; Penn State Hershey Flow Cytometry Core Facility). Data represent mean ± SD, *n* = 3 sample/genotype from 2 independent experiments.

### Trypan blue exclusion

Cells that were non-adherent 24 h post-transfection were collected, along with adherent cells and stained with 0.4 % trypan blue solution at a 1:2 dilution. Cell viability was determined by counting the number of blue cells with a hemocytometer and dividing that number by the total number of cells. Data represent mean ± SD, *n* = 3 samples/genotype from 3 independent experiments.

### Measurement of CDK2-associated kinase activity

Immunoprecipitation of CDK2 protein containing complexes and determination of associated kinase activity were both determined as previously described [67]. Briefly, protein extracts were prepared from 2 × 10^6^ MDA-MB-231 mock-transfected cells and cells over-expressing ZnT2 using the glass-bead breakage method, followed by immunoprecipitation of CDK2-containing protein complexes from 200 μg of protein, with the exception of the pre-clearing step which was performed using a 1:1000 dilution of normal rabbit IgG (Upstate). Immunoprecipitated CDK2 complexes and CDK2 protein expression levels in total cell lysates were determined using immunoblot analysis. Immunoprecipitated CDK2-containing protein complexes were subjected to a Histone H1 kinase assay. Briefly, immunoprecipitated CDK2 complexes were washed once in immunoprecipitation buffer and then twice with kinase reaction buffer. Kinase reactions were performed in a final volume of 20 μL consisting of kinase buffer supplemented with 20 μM ATP, 10 μCi of [γ-^32^P] ATP, and 1 μg of histone H1 (Roche) as substrate. Kinase assay mixtures were incubated at 30 °C for 30 min. Reactions were stopped with the addition of loading buffer, and samples were boiled for 10 min. Reactions were resolved by electrophoresis on a 10 % SDS-polyacrylamide gel and dried, followed by autoradiograpy.

### Apoptosis

Apoptosis was determined via the Annexin V-FITC kit (Trevigen, Gaithersburg, MD). Briefly, 3 × 10^5^ cells were transfected in a 6-well plate; 24 h later, cells were trypsinized (2 min), washed and centrifuged at 300 × g for 5 min at room temperature. Cells were stained with both Annexin V-FITC and propidium iodide for 15 min in the dark at room temperature. Cells were washed and processed by flow cytometry. Data represent mean ± SD, *n* = 3 samples/genotype from 3 independent experiments.

### Invasion assay

Cells were transfected as previously described and 4 × 10^4^ cells were added to 200 μL of serum- and antibiotic-free growth medium per Boyden insert while 600 μL of antibiotic-free growth medium, with 10 % FBS, was added to the bottom of the well (in a 24-well plate). Cells were incubated in a humidified chamber for 24 h at 37 °C. Inserts were removed and submerged in PBS several times to remove unattached cells. Non-invading cells were removed by gently scraping with a wet cotton applicator. Cells were fixed by submerging the inserts into 4 % paraformaldehyde for 10 min. The insert was washed with 1× PBS and stained with hematoxylin and 3 % glacial acetic acid for 30 min. The insert was washed gently, several times with distilled water. Migrated cells were visualized under light microscopy at 40× by cutting out the porous membrane of the insert and mounting on a slide (migrated side down). Data represent mean ± SD, *n* = 3 samples/genotype from 2 independent experiments.

### Statistical analysis

Gene names (*SLC30A* and *SLC39A*) and concomitant proteins (ZnT and ZIP, respectively) are used to differentiate between gene and protein expression. Results of studies in cultured cells are presented as mean ± SD or SEM where indicated. Statistical comparisons were performed using Student’s *t*-test or one-way ANOVA as indicated (Prism Graph Pad, Berkeley, CA). A significant difference was demonstrated at *p* < 0.05.

## Additional file

Additional file 1: Table S1. Summary of the molecular mass of Zn transporters and antibodies used for detection [68–87]. (DOCX 35 kb)

## Abbreviations

Ca: calcium
ER HG: estrogen receptor, high-grade
ER LG: estrogen receptor, low grade
ER^+^: estrogen receptor positive
ER^+^/PR^+^/HER2^+^: estrogen, progesterone, HER2 positive
ER^−^/PR^−^/HER2^−^: estrogen, progesterone, HER2 negative
FDR: false discovery rate
MREs: metal responsive elements
MT: metallothionein
PBS-T: phosphate buffered saline/tween-20
*SCL39A1-14*: solute carrier family 1–14
*SLC30A1-10*: solute carrier family 1–10
ZIP1-14: ZRT/IRT-like protein 1–14
Zn: zinc
ZnT1-10: zinc transporter 1–10
