# Supplementary material for: Subtype-specific accumulation of intracellular zinc pools is associated with the malignant phenotype in breast cancer
Source: Mol Cancer. 2016 Jan 5;15:2. doi: 10.1186/s12943-015-0486-y (PMC4700748; doi:10.1186/s12943-015-0486-y)
Supplement: Additional file 1: Table S1. — Summary of the molecular mass of Zn transporters and antibodies used for detection [68–87]. (DOCX 35 kb) [file 12943_2015_486_MOESM1_ESM.docx]

Additional file 1: Table S1. Summary of the molecular mass of Zn transporters and antibodies used for detection.

| **Transporter** | **Predicted MW^1^ (kDa)** | **Detected MW (kDa)** | **Reported MW (kDa)** | **Source** | **Reference** | **Antibody used** |
| --- | --- | --- | --- | --- | --- | --- |
| ZIP1 | 34 | 66, 68 | 66 | recombinant | Santa Cruz  (sc-15632) | Santa Cruz  (sc-15632) |
|  |  |  | 35, 48, 66 | TRAMPC2 cells | Costello et al, [[68](#_ENREF_68)] |  |
| ZIP3 | 34 | 66, 130 | 38 | mammary cells | Kelleher et al, [[50](#_ENREF_50)] | Kelleher et al, [[50](#_ENREF_50)] |
| ZIP4 | 66, 68 | 35, 52, 54 | 60 | CaCO2 cells | Jou et al, [[69](#_ENREF_69)] | JJou et al, [[69](#_ENREF_69)] |
|  |  |  | 35, 60, 71 | mouse yolk sac | Dufner-Beattie et al, [[70](#_ENREF_70)] |  |
|  |  |  | 73 | HEPA cells | Weaver et al, [[71](#_ENREF_71)] |  |
| ZIP5 | 56 | 56 | 58 | human spleen | ProSci (6089) | ProSci (6089) |
|  |  |  | 56 | mouse yolk sac | Dufner-Beattie et al, [[70](#_ENREF_70)] |  |
| ZIP6 | 48, 85 | 48 | 50, 84 | unknown | Sigma  (SAB3500597) | SSigma (SAB3500597) |
|  |  |  | 90 | MCF7 cells | Shen et al, [[72](#_ENREF_72)] |  |
|  |  |  | 42, 84 | mouse testes | Croxford et al, [[73](#_ENREF_73)] |  |
| ZIP7 | 50 | 72 | 56 | mouse brain, liver | Huang et al, [[35](#_ENREF_35)] | Huang et al, [[35](#_ENREF_35)] |
|  |  |  | 56, 58, 72, 105, >250 | TAMR breast cells | Taylor et al, [[37](#_ENREF_37)] |  |
| ZIP8 | 43, 49 | 52, 56 | 55 | human spleen | Pierce  (PA5-21073) | Pierce  (PA5-21073) |
|  |  |  | 55, 140 | BEAS-2B cells | Besecker et al, [[74](#_ENREF_74)] |  |
|  |  |  | 60, 62 | mouse RBCs | Ryu et al, [[75](#_ENREF_75)] |  |
|  |  |  | 75, 150 | human T-cells | Aydemir et al, [[76](#_ENREF_76)] |  |
| **Transporter** | **Predicted MW (kD)** | **Detected MW (kD)** | **Reported MW (kD)** | **Source** | **Reference** | **Antibody used** |
| ZIP10 | 94 | 47, 49 | 50, 95 | human spleen | Pierce (PA5-20678) | PPierce (PA5-20678) |
|  |  |  | 40 | mouse RBCs | Ryu et al, [[75](#_ENREF_75)] |  |
|  |  |  | 40, 42 | mouse testes | Croxford et al, [[73](#_ENREF_73)] |  |
| ZIP11 | 35 | 46, 52 | 35 | mouse kidney | Pierce  (PA5-20679) | Pierce  (PA5-20679) |
|  |  |  | 42, 44 | mouse mammary gland | Kelleher et al, [[50](#_ENREF_50)] |  |
| ZIP12 | 73,77 | 35 | 42 | unknown | Sigma  (SAB35006010) | Sigma (SAB35006010) |
|  |  |  | 35, 72 | mouse mammary gland | Kelleher et al, [[50](#_ENREF_50)] |  |
| ZIP14 | 52, 54 | 35, 52, 76 | 35, 52, 76 | U251 cells | Abcam (ab123988) | Abcam (ab123988) |
|  |  |  | 50 | mouse liver | Liuzzi et al, [[77](#_ENREF_77)] |  |
|  |  |  | 55 | HEPG2 cells | Gao et al, [[78](#_ENREF_78)] |  |
| ZnT1 | 55 | 52 | 50 | rat mammary gland | Kelleher et al, [[66](#_ENREF_66)] | Kelleher et al, [[66](#_ENREF_66)] |
|  |  |  | 35 | mouse RBCs | Ryu et al, [[75](#_ENREF_75)] |  |
|  |  |  | 38, 52 | mouse liver | Yu et al, [[79](#_ENREF_79)] |  |
| ZnT2 | 35, 41 | 42 | 35, 42 | mammary cells | Lopez et al, [[45](#_ENREF_45)] | Lopez et al, [[45](#_ENREF_45)] |
|  |  |  | 42 | mouse pancreas | Guo et al, [[80](#_ENREF_80)] |  |
| ZnT3 | 36 | 52 | 48 | SK-N-SH cells | Santa Cruz  (sc-27508) | Santa Cruz  (sc-27508) |
|  |  |  | 45 | hamster kidney | Palmiter et al, [[81](#_ENREF_81)] |  |
| **Transporter** | **Predicted MW (kD)** | **Detected MW (kD)** | **Reported MW (kD)** | **Source** | **Reference** | **Antibody used** |
| ZnT4 | 48 | 42, 48 | 48 | mouse mammary gland | Kelleher et al, [[50](#_ENREF_50)] | Kelleher et al, [[50](#_ENREF_50)] |
|  |  |  | 45 | CaCO2 cells | Murgia et al, [[82](#_ENREF_82)] |  |
|  |  |  | 47,77 | PMC42 breast cells | Michalczyk et al, [[83](#_ENREF_83)] |  |
| ZnT5 | 55, 57, 84 | 48, 52 | 68 | PaCa-2 cells | Santa Cruz  (sc-161272) | Santa Cruz  (sc-161272) |
|  |  |  | 55 | HEp-2, Hep3B, JAR, and HeLa cells | Kambe et al, [84] |  |
|  |  |  | 55, 57 | human breast tissue, PMC42 breast cells | Kumar et al, [[85](#_ENREF_85)] |  |
|  |  |  | 87 | RWPE2 cells,  mouse small intestine and liver | Yu et al, [[79](#_ENREF_79)] |  |
| ZnT6 | 47, 49, 51, 56 | 47, 55, 110 | 55 | mouse lung | Huang et al, [[86](#_ENREF_86)] | Huang et al, [[86](#_ENREF_86)] |
| ZnT8 | 41 | 49, 53 | 41 | Raji cells, mouse spleen | Abbiotec (254333) | Abbiotec  (254333) |
| ZnT9 | 64 | 63 | 60 | HEPG2 cells | Santa Cruz  (sc-134214) | Santa Cruz  (sc-134214) |
| ZnT10 | 53 | 100 | 50 | ZR-75-1 cells | Abcam (ab139058) | Abcam (ab139058) |
